# Is aclidinium alone or combined with a LABA a rational choice for symptomatic COPD patients?

**DOI:** 10.1186/s12931-017-0506-0

**Published:** 2017-01-18

**Authors:** F. Blasi, G. W. Canonica, M. Miravitlles

**Affiliations:** 1 Department of Pathophysiology and Transplantation, Università degli Studi di Milano, Cardio-thoracic unit and Cystic Fibrosis Adult Center Fondazione IRCCS Cà Granda Ospedale Maggiore Policlinico Milano, Milan, Italy; 2 Department of Biomedical Science, Personalized Medicine Clinic: Asthma & Allergy - Humanitas Clinical and Research Center, Humanitas University –Rozzano (Milano), Milan, Italy; 3 Pneumology Department, Hospital Universitari Vall d’Hebron, Barcelona, Spain

**Keywords:** COPD, Bronchodilators, Symptoms, Circadian variability, Therapeutic control, LAMA, Aclidinium, LAMA + LABA, Aclidinium + formoterol

## Abstract

**Background:**

As emphasized by international recommendations and largely confirmed by clinical experience, long-acting bronchodilators play a central role in the maintenance treatment of chronic obstructive pulmonary disease (COPD) due to their proven efficacy in reducing airflow obstruction and improving symptoms.

**Main body:**

There are some important aspects to define with regard to inhalation therapy for COPD, particularly those concerning the selection criteria and the optimal use of long-acting bronchodilators. First of all, it needs to be determined in which patients and clinical situations monotherapy with one bronchodilator, such as a long-acting muscarinic antagonist (LAMA), should be considered adequate, and in which cases the use of combination therapies, such as the “double bronchodilation” with a LAMA and a long-acting β2-agonist (LABA), should be preferred. Another critical issue concerns the effect of the frequency of daily administration of inhaled agents on the control of symptoms during the 24 h. COPD symptoms are known to exhibit considerable circadian variability with worsening in the early morning, and a significant proportion of patients have disease-related sleep disorders which can adversely affect their quality of life. The worsening of symptoms in the early morning may be due, at least in part, to a reduction in airway caliber caused by an increased “cholinergic tone” at night. As such, the coverage of nighttime and early morning symptoms is a reasonable therapeutic goal, which can be achieved by many patients using LAMAs such as aclidinium bromide twice daily (BID). Therapeutic adherence is known to be a multifactorial phenomenon that is frequently affected by other aspects than dosing frequency, including the technical features and ease of use of the inhalers. To this end, it should be mentioned that certain new-generation inhalers such as Genuair® have been associated in clinical trials with higher patient preference.

**Conclusion:**

In this work, in addition to presenting an overview of the main evidence on the efficacy of COPD treatment with the LAMA aclidinium bromide BID, we suggest some selection criteria for the monotherapy with one long-acting bronchodilator or the combination therapy with LAMA and LABA in COPD patients, with particular reference to specific clinical scenarios.

## Background

Chronic obstructive pulmonary disease (COPD) is a common, preventable and treatable disease characterized by persistent, usually progressive airflow limitation, caused by both small airway disease (obstructive bronchiolitis) and parenchymal destruction (emphysema), the relative contributions of which vary from person to person. Characteristic symptoms of this chronic respiratory disease include dyspnea, cough and sputum production. Usually, a person suffering from COPD will decide to consult a physician because of the negative impact of their symptoms on their daily living activities, but also because of the persistence of symptoms or the occurrence of exacerbations.

COPD, a major cause of morbidity and mortality worldwide, involves a significant and increasing economic and social burden. The disability adjusted life years (DALYs) for a specific disease are calculated as the sum of the years of life lost due to premature mortality and the years of life lived with disability, adjusted for the severity of the disease. In 1990, COPD was the twelfth leading cause of lost DALYs worldwide, accounting for 2.1% of the total. It is predicted that by 2030 this disease will be the seventh leading cause of lost DALYs globally [1].

## Main text

### Direct and indirect costs of COPD

COPD involves a significant economic burden. In the European Union, the total direct costs of respiratory disease are estimated to be about 6% of the total health budget, with COPD alone accounting for 56% (38.6 billion Euros) of this budget. In the United States, the costs of COPD are estimated to be $29.5 billion for direct costs and $20.4 billion for indirect costs. Exacerbations account for the greatest proportion of health care costs related to COPD. Therefore, the close correlation between disease severity and health care costs is not surprising, with the distribution of health expenditure changing with the progression of the disease. Any estimate of the direct medical costs of home care does not reflect the true home care costs for society, as it does not take into account the economic value of care provided by family members to COPD patients [2].

### Pathophysiology of COPD symptoms - circadian variability

The traditional definition of COPD as a slowly progressive disease in which the deterioration of lung function is associated with an increase in symptoms, has been questioned by scientific evidence accumulated in recent years. Several studies have in fact demonstrated that the extent and perception of COPD symptoms are not as stable as previously believed, but show a variability that is not only seasonal, but also weekly and even daily, with symptoms often worsening at night and in the early morning [3, 4].

Circadian variability of COPD symptoms was also confirmed by a survey conducted on a sample of 803 COPD patients, which showed an increase in clinical manifestations especially in the early morning (*P* <0.001 versus “midday”, “afternoon”, “evening”, “night” and “difficult to say” for all COPD patients; *P* <0.001 versus “midday” for patient with severe COPD symptoms) and at night [5]. There is also evidence that the main symptoms of COPD show different temporal trends during the 24 h; in particular, it has been found that dyspnea, sputum production and cough tend to worsen especially in the morning, while wheezing shows a peak of variability at night, and tightness in the chest displays a highly variable trend during the 24 h [6].

From the pathophysiological viewpoint, the circadian variability of COPD symptoms, and especially the increase in symptoms at night/early morning, may be due, at least in part, to circadian modulation of the airway caliber by the cholinergic system. Like many other biological variables, airway caliber shows a certain variability during the 24 h, reaching peak values around midday, with lower values at night and in the early morning. A central cholinergic mechanism is believed to be responsible for modulating the variability in airway caliber (“cholinergic tone”). The worsening of symptoms at night or in the early morning, which is found in many patients with COPD, may be at least partially attributed to a pathological increase in this circadian variation [7].

### Nighttime/early morning symptoms and decreased performance/QoL in COPD patients

It is currently believed that improvements in “patient-centered” outcomes, including subjective symptoms, can reflect more accurately the effectiveness of pharmacological treatment of COPD compared with changes in forced expiratory volume in 1 s (FEV1), which are often transient and do not adequately reflect the real impact of treatment on the quality of life of patients.

A weak correlation between symptom perception by patients and FEV1 values is, in fact, often found in COPD; accordingly, subjective indicators relating to quality of life (QoL) and health-related quality of life (HR-QoL) are now considered as part of the therapeutic evaluation [8]. An example of such “patient-centered” outcomes is given by the circadian variability of COPD symptoms, which has been shown to adversely affect the performance of normal daily activities and to have a substantial negative impact on the quality of life [4].

Nighttime symptoms are particularly troublesome for patients with COPD as these symptoms are often associated with poor quality of sleep [9, 10]. However, the presence of nocturnal symptoms in a patient with chronic airway disease should prompt health care professionals to make a differential diagnosis between COPD and asthma, as well as to exclude or confirm the presence of (concomitant) heart failure. To this end, a survey conducted by primary care physicians and respiratory specialists from several European countries (France, Germany, Italy, Spain and the UK) on a retrospective cohort of 2807 patients with COPD, showed that the majority of enrolled subjects (78%) reported the presence of sleep disorders (i.e., difficulty in falling asleep, frequent nocturnal awakenings, difficulty in maintaining sleep), with nighttime symptoms associated with greater disease severity i.e., more daytime symptoms and more frequent exacerbations) [11, 12].

Similar to effects of nighttime symptoms, various studies have found that morning symptoms are also a significant burden to patients with COPD. Moreover, early morning has been reported as the worst time of a day by many COPD patients, especially among those with severe disease; moreover, early morning COPD symptoms limit patients’ ability to perform their normal activities [13–15].

The recent observational ASSESS study, which included more than 700 patients with COPD, performed in a real-life context, has confirmed the significant prevalence of nighttime symptoms (52%), which tend to be associated both with greater symptom severity, as assessed by the COPD Assessment Test (CAT), and higher prevalence of anxiety and depression, as assessed by the Hospital Anxiety and Depression Scale (HADS) [16]. In the clinical practice, however, the circadian variability is often not adequately taken into consideration in the therapeutic approach; for example, a real-life clinical study showed that drug treatment was not changed in more than half of patients surveyed, even though a significant worsening of symptoms over 24 h was noted. On the other hand, paying attention to the progression of symptoms over the course of 24 h is also important from the therapeutic point of view, as this can help to identify different types of patients, as well as to establish the most appropriate therapeutic strategy to mitigate the impact of symptoms on HR-QoL.

For these reasons, patients with COPD should be specifically questioned about daytime and nighttime symptoms and their impact on quality of life. The implementation of appropriate strategies for the optimal control of symptoms is also recommended, with particular regard to the time of maximum symptom intensity (e.g., nighttime, early morning) [4].

### Inappropriate overuse of LABA + ICS and LABA + LAMA + ICS combinations in COPD patients that belong to A or B-GOLD groups

Although the Global strategy for the diagnosis, management, and prevention of COPD (GOLD) recommendations indicate that monotherapy with a long-acting bronchodilators is the treatment of choice in group A and B patients, an inappropriate “overuse” of combinations with inhaled corticosteroids (ICS) and bronchodilators is often observed in the clinical practice. This inappropriate overuse of ICS-containing regimens is often in conflict with the need for a careful real-world assessment of the risk-benefit ratio of COPD treatments [17].

**Fig. 1:**
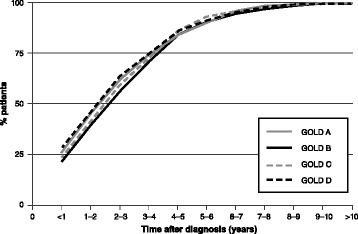
Cumulative proportion of COPD patients receiving “triple therapy” (TT) with LABA + LABA + ICS by GOLD (Global initiative for chronic Obstructive Lung Disease) group, based on a UK primary-care database (2002–2010). Note: *P* = 0.065 (chi-square test). Elaboration of textual data from [16]

To this end, it is worth mentioning the results of a recent observational study conducted in the UK on a dataset of primary-care patients with COPD, which showed that the prescription pathways leading to a real-world use of “triple therapy” (TT) with LABA + LAMA + ICS differ considerably from those recommended by international recommendations [18]. In particular, this study found that ICS-containing TT regimens were also frequently prescribed to patients at low-risk of exacerbations and/or without concomitant asthma, who, according to current guidelines, do not need such treatments. The “progression” to TT occurred in 25% of cases within the first year of treatment and in 40% within the first two years [18] (Fig. 1).

The overuse of ICS-containing regimens can lead to several problems from the point of view of public health, with increased costs not justified by any superior efficacy, and from the viewpoint of safety, since chronic exposure to ICS can carry the risk of several side effects (including pneumonia, osteoporosis, etc) [2, 18]. Another fact emerging from the above UK survey is the low prescribing rates of long-acting bronchodilators as first-line therapy, which, together with the evidence of inappropriate prescribing of TT, emphasizes the need to provide better information and training for health care providers, in order to improve real-world implementation of COPD recommended management programs [18–22].

### Rationale of bronchodilator monotherapy in symptomatic COPD patients

The administration of long-acting bronchodilators, such as LAMA or LABA, plays a central role in the control of symptomatic COPD [16]. There is also evidence showing that bronchodilators may have a significant role in the control of circadian variability of symptoms and in improving the quality of life of patients with COPD [4].

An interesting therapeutic “target” in this context is the increased “cholinergic tone” in patients with COPD; this phenomenon, due to various mechanisms not yet fully elucidated [23], seems to be particularly relevant at night and may contribute to the frequent occurrence of early morning symptoms in patients with COPD. The therapeutic role of LAMAs is therefore of great interest in view of these pathophysiological aspects, which are particularly important in patients with nocturnal symptoms and/or significant circadian variability of symptoms. The effectiveness of these drugs in reducing airway obstruction and lung hyperinflation, leading to an improvement in a variety of “patient-centered” outcomes, is well-documented [24]. In particular, due to their specific modulating action on cholinergic tone, LAMAs can effectively control the symptoms for 24 h, especially when administered as a twice-daily regimen, which provides a better coverage of the “critical” nighttime period, as in the case of aclidinium bromide [25].

### Role of the LAMA aclidinium bromide in COPD treatment

Aclidinium bromide is a potent and selective LAMA, characterized by rapid onset and long duration of action; it also has a good cardiovascular safety profile, which may be attributed to its reduced residence time at M2 receptors and its rapid hydrolysis in plasma [26].

In experimental models, aclidinium showed a faster onset of action than the LAMA tiotropium [26]. Furthermore, in a study that assessed the onset of action of LAMAs (aclidinium, glycopyrronium and tiotropium, all administered at the approved clinical doses) in moderate-to-severe COPD patients, bronchodilation induced by aclidinium and glycopyrronium was faster than that induced by tiotropium, without significant difference between aclidinium and glycopirronium [27].

Aclidinium bromide also exhibits potential anti-inflammatory and anti-airway remodeling effects, as shown by studies of experimental animal models, which can be an “added value” in the therapeutic management of COPD [23].

For example, there is some evidence that aclidinium bromide could modulate the non-neuronal cholinergic system, a recently identified regulatory pathway in pulmonary inflammation and remodeling [28]. Dysfunction of the non-neuronal cholinergic system seems to be involved in the pathophysiology of COPD and to some extent in resistance to corticosteroid therapy; in fact, non-neuronal cholinergic system is over-expressed in corticosteroid-insensitive neutrophils from COPD patients, as evidenced by increases in the expression of muscarinic receptors (M2, M4 and M5), vesicular acetylcholine transporter (VAChT) and choline acetyltransferase (ChAT).

In a recent in vitro study, aclidinium bromide demonstrated anti-inflammatory effects on neutrophils from COPD patients, reversing their resistance to corticosteroids; in fact, the addition of aclidinium bromide increased the impaired anti-inflammatory properties of a corticosteroid (fluticasone propionate) by a mechanism involving the inhibition of Glucocorticoid Receptor Alpha (GRα) phosphorylation at Ser-226, the enhancement of corticosteroid- mediated Glucocorticoid Response Element (GRE) activation and the expression of corticosteroid-dependent anti-inflammatory genes, i.e. Mitogen-activated Protein Kinase Phosphatase 1 (MKP1), Cysteine-RIch Secretory Protein LCCL Domain-containing 2 (CRISPLD2) and (GILZ) [28]. Among aclidinium bromide therapeutic properties, it seems to be interesting as well its favourable effect on hyperinflation and airflow limitation, that plays a very important pathophysiological role in inducing dyspnea and reducing quality of life (QoL) in COPD patients; in this regard, it has been recently demonstrated that aclidinium promotes a rapid and relevant desufflation and improves lung ventilation inhomogeneity, even in severe/very severe COPD patients [29].

The clinical efficacy of twice-daily (BID) aclidinium bromide is clearly demonstrated by the results of a large number of both placebo-controlled and active-comparator trials [30–36].

With regard to overall efficacy in the treatment of COPD, a Cochrane Review of 12 double-blind randomized controlled trials, lasting 4 to 52 weeks, on a total of 9547 patients with stable COPD, demonstrated that aclidinium bromide BID significantly improved quality of life by lowering the St. George’s Respiratory Questionnaire (SGRQ) total score by 2.34 units compared to placebo (95% confidence interval [CI] 3.18 to 1.51), significantly increased FEV1 with a mean difference of 0.09 L compared to placebo (95% CI 0.08 to 0.10), and reduced the number of patients with exacerbations requiring hospitalization (OR 0.64; 95% CI 0.46 to 0.88) [31].

Aclidinium bromide BID can provide sustained bronchodilation over 24 h, which is associated with significant improvement in FEV1 area under the curve (AUC), especially at night and early morning [32].

As regards the potential to effectively control the circadian variability of COPD symptoms, particularly interesting are the results of a multicenter, randomized, double-blind trial comparing aclidinium bromide 400 μg BID with placebo and tiotropium 18 μg OD in 795 patients with moderate-to-severe COPD, treated in a real-life context. During the period of treatment with aclidinium bromide 400 μg BID, the proportions of patients with nighttime and morning symptoms and limitation in morning activities were significantly reduced from baseline (*p* <0.0001 for all tests) [33].

The efficacy of aclidinium bromide in terms of symptom control over the entire 24-h period was also confirmed in a double-blind, randomized trial comparing aclidinium 400 μg BID with tiotropium 18 μg once-daily (OD) or placebo for six weeks on 414 patients with moderate-to-severe COPD [30]. At the end of the treatment period, symptom scores, assessed using specific questionnaires, were significantly reduced from baseline with both aclidinium (*p* <0.0001) and tiotropium (*p* <0.05) compared to placebo. It should be noted, however, that only aclidinium, but not tiotropium, resulted in significant improvements compared with placebo with regard to individual morning symptoms (phlegm, shortness of breath, wheezing, and cough) and severity of nocturnal symptoms; moreover, only aclidinium significantly reduced the limitation of activity caused by symptoms, with a significant difference compared to tiotropium (*p* <0.05). The results of this study, in terms of greater potential of aclidinium to control the symptomatic manifestations of COPD, might be related to differences in dosing frequency between aclidinium and tiotropium; in particular, the second evening dose of aclidinium, administered in the evening, which is closer to the time of maximum intensity of respiratory symptoms, may be beneficial in improving nighttime and early morning symptoms.

In COPD patients, reducing the severity of respiratory symptoms is a very important treatment goal, as they are associated with poor health outcomes, reduced health status and increased exacerbation risk.

In this regard, a recent post-hoc analysis of pooled data from the aclidinium 400 μg BID and placebo arms of two 24-week, double-blind, randomized studies, evaluating aclidinium monotherapy or combination therapy with aclidinium plus LABA formoterol, assessed the effect of aclidinium bromide on respiratory symptoms in moderate-to-severe COPD patients. According to the conclusions of this paper, aclidinium 400 μg BID significantly improved COPD respiratory symptoms, as assessed by the Evaluating Respiratory Symptoms in COPD (ERS-COPD) scale, irrespective of the patients’ level of symptoms at baseline; in fact, net treatment benefit by aclidinium compared to placebo was found to be similar in patients with low or high levels of COPD symptoms [37].

Aclidinium bromide has confirmed its efficacy also in the long term, as demonstrated by the results of a 52-week double-blind, parallel group trial, performed on 605 patients with moderate-to-severe COPD. Treatment with aclidinium bromide BID resulted in clinically important improvements (reduction ≥4 points from baseline) in symptom scores and in quality of life as assessed by SGRQ score at all study visits (performed at weeks 1, 12, 24, 36, 48 and 52) [34].

With regard to the tolerability profile, it should be noted that receptor selectivity and rapid plasma hydrolysis of aclidinium to inactive metabolites can explain the low incidence of systemic adverse events observed in clinical trials; such events, including anticholinergic side effects, were also generally mild [35, 36] (Table 1).

**Table 1:** Number (%) of patients with potential anticholinergic adverse events by system organ class and preferred term (safety population) (*Table 3 of* [36], reproduced by permission)

System organ class/preferred term	Placebo (*n* = 182)	Aclidinium 200 μg (*n* = 183)	Aclidinium 400 μg (*n* = 177)
Cardiac disorders			
Tachycardia	0	0	1 (0.6)
Arrhythmia	1 (0.5)	0	0
Bradycardia	2 (1.1)	0	0
Palpitations	0	2 (1.1)	0
Increased heart rate^a^	0	2 (1.1)	0
Eye disorders			
Transient blindness	0	1 (0.5)	0
Reduced visual acuity	0	1 (0.5)	0
Gastrointestinal disorders			
Constipation	3 (1.6)	1 (0.5)	0
Dry mouth	1 (0.5)	2 (1.1)	3 (1.7)
Infections and infestation disorders			
Urinary tract infection	0	3 (1.6)	1 (0.6)
Nervous system disorders			
Optic neuritis	0	0	1 (0.6)
Renal and urinary disorders			
Urinary retention	0	0	1 (0.6)
Urinary incontinence	1 (0.5)	0	0

^a^Investigations is the system organ class for this preferred term

As a result of the large systemic and pre-systemic hydrolysis, the absolute bioavailability of aclidinium is very low (<5%), and significantly lower than that of tiotropium and glycopyrronium (45% and 19%, respectively) [38–40]. Cardiovascular adverse events have been reported with similar frequency in patients treated with aclidinium bromide and in those assigned to placebo group, supporting the safety of aclidinium in patients with COPD and concomitant cardiovascular disease [41, 42].

### Adherence to inhaled therapies: what is more important, frequency of administration or patient preference?

Some authors have advanced the hypothesis that, in terms of patient’s adherence to medical treatment, twice-daily dosing of inhaled medications is less favorable than once-daily dosing, but the available evidence in literature does not support this hypothesis. For instance, an observational study has demonstrated that the high frequency of drug administration may be a major problem for therapeutic adherence only in the case of three- and four-times-daily regimens, while there are no particularly significant differences between twice- and once-daily regimens [31].

On this subject there are interesting data from a recent study conducted in Spain on more than 16,000 patients treated with inhaled LAMAs (aclidinium, tiotropium, or glycopyrronium), which evaluated the degree of adherence to two different dosing regimens (once- or twice-daily).

This study showed that adherence to treatment with LAMAs was generally very high, irrespective of the molecule or inhalation device used; in particular, there was no evidence of lower adherence to LAMAs with BID dosing compared with OD dosing [43].

In this regard, an intriguing hypothesis, which needs to be verified in future studies, is that the better control of symptoms (especially of nighttime and early-morning symptoms) associated with BID dosing may be a significant factor for improving treatment adherence to this dosing regimen.

It should also be remembered that therapeutic adherence is a multifactorial process that can be affected by a number of factors different from dosing frequency, including: perceived efficacy of treatment, side effects, number of concomitant medications, as well as patient’s satisfaction with, or preference for, the inhaler device [31].

With regard to the latter, it should be emphasized that, in order to achieve adequate adherence and, thus, the maximum benefit from treatment, it is important that the inhalation device is used correctly. Since an inadequate inhaler technique may compromise treatment efficacy, it is desirable to have devices that are easy to use, with a few simple “steps” for dosing, and with feedback mechanisms to confirm successful inhalation of the dose. An example of this kind of device is Genuair®, a multidose, breath-actuated dry powder inhaler (DPI), designed to deliver aclidinium bromide. Genuair® incorporates a number of technological features that enhance its performance and safety of use, including the patented “cyclone” technology to improve deaggregation of aerosol particles, thus achieving high drug deposition in the lungs, as well as visual and acoustic feedback mechanisms that make the device simple and easy-to-use for the patient [44].

**Fig. 2:**
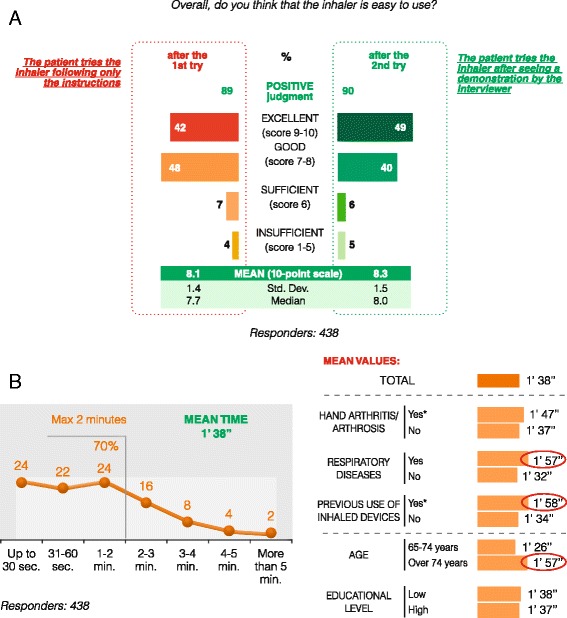
Assessment of ease of use of the device (Panel **a**) and mean time to learn the inhaler technique and to perform a correct inhalation (Panel **b**), as reported by a recent Italian study which evaluated the usability of the Genuair® device in a representative sample of the elderly population with COPD/hand arthritis/arthrosis. Elaboration of textual data from [45]

Ease of use, efficiency, and low probability of critical errors with Genuair® are clinically reflected in an increased patient preference for this inhaler compared to other frequently used devices. To this end, in a recent Italian study investigating the “usability” of this device in a representative sample of the elderly population (256 individuals with COPD, and 89 with hand arthritis/arthrosis), Genuair® was considered a well-accepted, easy-to-use device by the great majority of patients studied; moreover, the mean time to learn and perform the inhalation correctly was only 1’38”, and more than 70% of patients took less than 2 min to perform this task [45] (Fig. 2).

### Aclidinium/formoterol: rationale of combination for clinical use in COPD patients

The long-acting bronchodilators, LAMA and LABA, play an important role in the treatment of COPD, as emphasized by international recommendations [2, 46, 47]. The combined use of bronchodilators belonging to different drug classes, acting on different receptor systems, can improve lung function and symptoms, which is particularly beneficial in patients with moderate-to-severe COPD [41].

The rationale of the “double bronchodilation” with LAMA and LABA lies in the fact that the relaxation of airway smooth muscle (and the resulting bronchodilation) can be achieved by two main mechanisms:

- inhibition of acetylcholine signaling, mediated by muscarinic M3 receptors, on airway smooth muscle with LAMA;
- stimulation of β2-adrenergic receptors with LABA.

The simultaneous pharmacological targeting of these two mechanisms of bronchodilation can maximize the bronchodilator response, without the need to increase the dose of each component of the combination and thereby reduces the risk of adverse events [48–50].

Fixed-dose combinations (FDCs) of LAMA and LABA offer the potential of good convenience and compliance. Among the different LAMA/LABA FDCs, the combinations involving twice-daily dosing, such as the LAMA aclidinium + LABA formoterol, may be able to better tailor drug therapy to the individual needs of patients, including the control of early-morning symptoms, which are particularly intense and/or frequent in patients with moderate-to-severe COPD [51].

This was confirmed by a pooled analysis of two 24-week, double-blind, placebo-controlled, randomized phase III trials evaluating the efficacy of the FDC aclidinium/formoterol on 3394 patients with moderate-to-severe COPD. This analysis showed that the FDC aclidinium/formoterol was able to improve early morning symptoms and to reduce the limitation of early-morning activities significantly more (*p* <0.05) than placebo and monotherapy with LAMA or LABA [42].

### The correct use of bronchodilators in COPD, “when, and to whom”: therapeutic suggestions

The current availability of a number of active agents and a variety of drug classes for the treatment of COPD makes it often difficult for the physician to select the optimal treatment. In our view, the selection of the most appropriate treatment should take into consideration the extent of the “disease impact” in a given patient, including the severity of spirometric obstruction, the symptom burden, the frequency of exacerbations and disease control [48]. Although LAMA monotherapy may be adequate to control the disease in certain groups of patients with COPD, combined therapy with LAMA and LABA, in combination with an ICS if needed, is often necessary for specific clinical situations or certain levels of disease severity.

**Fig. 3:**
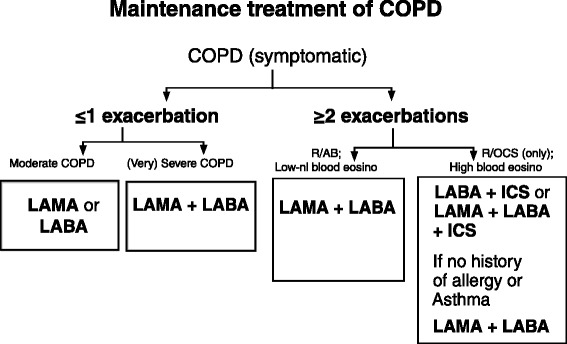
Therapeutic suggestions in COPD, taking also into account results of recent FLAME trial and the post-hoc analysis of the WISDOM trial. Elaboration of textual data from [51, 52]

What is nowadays the therapeutic role in COPD patients of LAMA used in monotherapy and/or in combination with other classes of inhaled drugs, such as LABA or ICS? We advance some therapeutic suggestions, taking also into account results of the recent FLAME trial and the post-hoc analysis of the WISDOM trial [51, 52] [(Fig. 3)]:

- COPD patients with ≤ 1 exacerbation/year and baseline FEV1 ≥ 50% predicted, first-line treatment with LAMA or LABA; baseline FEV1 < 50% and/or severe symptoms of dyspnea, first line treatment LAMA/LABA.
- COPD patients with ≥ 2 exacerbation/year and low eosinophils blood count [<300/μl], first-line treatment with LAMA/LABA; high blood eosinophil count [≥300/μl], first line treatment with LABA/ICS or LAMA/LABA/ICS; despite a high eosinophil count, LAMA/LABA can be considered first line treatment in absence of allergic or asthma history.

In case of lack of control a step-up therapy should be considered.

In any case it should be taken into account that the literature evidence suggests that in patients with mild airflow obstruction according to the GOLD spirometric classification and relatively moderate symptoms, LAMA monotherapy (for example, with aclidinium bromide; other LAMA commercially available are glycopyrronium bromide, tiotropium and umeclidinium) is often sufficient to provide adequate symptom control. In the same type of patients, but with more significant symptoms, the use of “double bronchodilation” with LAMA/LABA FDC (such as aclidinium + formoterol; other LAMA/LABA FDC commercially available are indacaterol/glycopyrronium, olodaterol/tiotropium, vilanterol/umeclidinium) can be considered. This LAMA/LABA is also preferred in patients with moderate-to-severe “disease impact”, including patients with moderate-to-severe airflow limitation and significant symptoms; patients with moderate-to-severe disease, who remain symptomatic despite monotherapy; patients with moderate-to-severe symptoms, but without frequent exacerbations. The use of a LAMA can be also considered as add-on therapy to LABA or ICS/LABA in case of very severe “disease impact”, e.g., in patients with severe spirometric obstruction suffering from frequent exacerbations. In these patients, with high eosinophil count but without history of allergy and asthma, a LAMA/LABA combination can be proposed as first choice.

## Conclusion

Bronchodilator therapy with LAMA, as monotherapy or in combination with LABA, is one cornerstone of COPD treatment. Given the circadian variability of symptoms in patients with COPD, due at least in part to the typical increased “cholinergic tone” in these subjects, the coverage of nighttime and early-morning symptoms should be considered a reasonable therapeutic target, which can be achieved by using an appropriate treatment (e.g. a LAMA such as aclidinium bromide, administered twice daily). Therapeutic adherence is often good in patients treated with twice-daily LAMAs, especially if they use a simple and easy-to-use inhalation device. The selection of LAMA monotherapy, combination therapy with LAMA + LABA, or add-on therapy with LAMA to LABA/ICS, should be individualized based on a comprehensive evaluation of the patient with COPD, taking into account the degree of airway obstruction, the symptom burden, eosinophil count, history of allergy and asthma and the frequency and type of exacerbations.

## Abbreviations

BID: Bis in die (i.e. twice daily)
CAT: COPD assessment test
ChAT: Choline acetyltransferase
CI: Confidence interval
COPD: Chronic obstructive pulmonary disease
CRISPLD2: Cysteine-rich secretory protein LCCL domain-containing 2
DALY: Disability adjusted life years
ERS-COPD: Evaluating Respiratory Symptoms in COPD
FDC: Fixed dose combination
GILZ: Glucocorticoid-induced leucine zipper
GOLD: Global strategy for the diagnosis, management, and prevention of COPD
GRE: Glucocorticoid response element
GRα: Glucocorticoid receptor alpha
HADS: Hospital Anxiety and Depression Scale
HR-QoL: Health-related quality of life
ICS: Inhaled CorticoSteroid
LABA: Long-acting β2-agonist
LAMA: Long-acting muscarinic antagonist
MKP-1: Mitogen-activated protein kinase phosphatase 1
OCS: Oral CortiCosteroid
OD: Once daily
QoL: Quality of life
SGRQ: St. George’s Respiratory Questionnaire
TT: Triple therapy
VAChT: Vesicular acetylcholine transporter
