# Erratum to: Is aclidinium alone or combined with a LABA a rational choice for symptomatic COPD patients?

**DOI:** 10.1186/s12931-017-0516-y

**Published:** 2017-02-10

**Authors:** F. Blasi, G. W. Canonica, M. Miravitlles

**Affiliations:** 1 Department of Pathophysiology and Transplantation, Università degli Studi di Milano, Cardio-thoracic unit and Cystic Fibrosis Adult Center Fondazione IRCCS Cà Granda Ospedale Maggiore Policlinico Milano, Milan, Italy; 2 Department of Biomedical Science, Personalized Medicine Clinic: Asthma & Allergy - Humanitas Clinical and Research Center, Humanitas University – Rozzano (Milano), Milan, Italy; 3 Pneumology Department, Hospital Universitari Vall d’Hebron, Barcelona, Spain

## Erratum

In the original publication of this article [1], the funding section omitted the following information: This work was funded by Momento Medico srl, which has also contributed to the costs for the literature selection and editorial assistance.
